# Bizarre Parosteal Osteochondromatous Proliferation: Nora's Lesion

**Published:** 2011-09-25

**Authors:** S. Chaabane, M. Chelli Bouaziz, K. H. Ben Ghars, L. Abid, M. H. Jaafoura, M. F. Ladeb

**Affiliations:** 1 Department of Radiology, Institut Kassab d'orthopédie, Ksar Said, Tunisia; 2 Professor, Department of Radiology, Institut Kassab d’orthopédie, Ksar Said, Tunisia; 3 Department of Pathology, Institut Kassab d’orthopédie, Ksar Said, Tunisia; 4 Professor, Department of Pathology, Institut Kassab d’or thopédie, Ksar Said, Tunisia

**Keywords:** Parosteal Osteochondroma, Bone, Radiography, Ultrasound, CT, MRI

## Abstract

The purpose of this study was to review the imaging and anatomopathologic findings and to discuss the main differential diagnosis of bizarre parosteal osteochondromatous proliferation (BPOP) or Nora's lesion, a rare benign surface lesion of the bone. Histologically confirmed plain radiographs, ultrasound, CT and MRI images of four patients with BPOP were obtained and retrospectively reviewed. Three cases involving the hand and one involving the foot are reported. On plain radiographs, BPOP is a wellmarginated, calcified or ossified mass arising directly from the cortical surface of the underlying bone. Ultrasound images show a low echoic peripheral cap around the lesion. CT images show the wide base of the lesion. On MRI, BPOP was of a low signal on T1, enhancing following gadolinium administration. Underlying bone and adjacent surrounding soft tissues were normal.

## Introduction

Bizarre parosteal osteochondromatous proliferation (BPOP) or Nora's lesion [1] was named by the pathologist who first described it in 1983 at the Mayo Clinic. He reported 35 lesions, all involving the small bones of the hands and feet. In 1993, Meneses et al.[2] reported 65 cases of BPOP involving various other sites. Further publications are limited to single cases or very small series.[3] To our knowledge, less than 170 cases of BPOP have been reported to date in the world literature. BPOP is a benign lesion that may be confused with other benign and malignant conditions. We present four cases of BPOP outlining clinical, radiological and histological findings. A review of the literature on BPOP as well as the different etiologic theories have been mentioned.

## Case Presentation

Case 1: A 52-year-old man presented with a five-year history of a mass on the palmar aspect of the right index finger. He claimed to have no history of trauma. On examination, the mass was hard, indolent and located at the level of the middle phalanx.

**Fig. 1:**
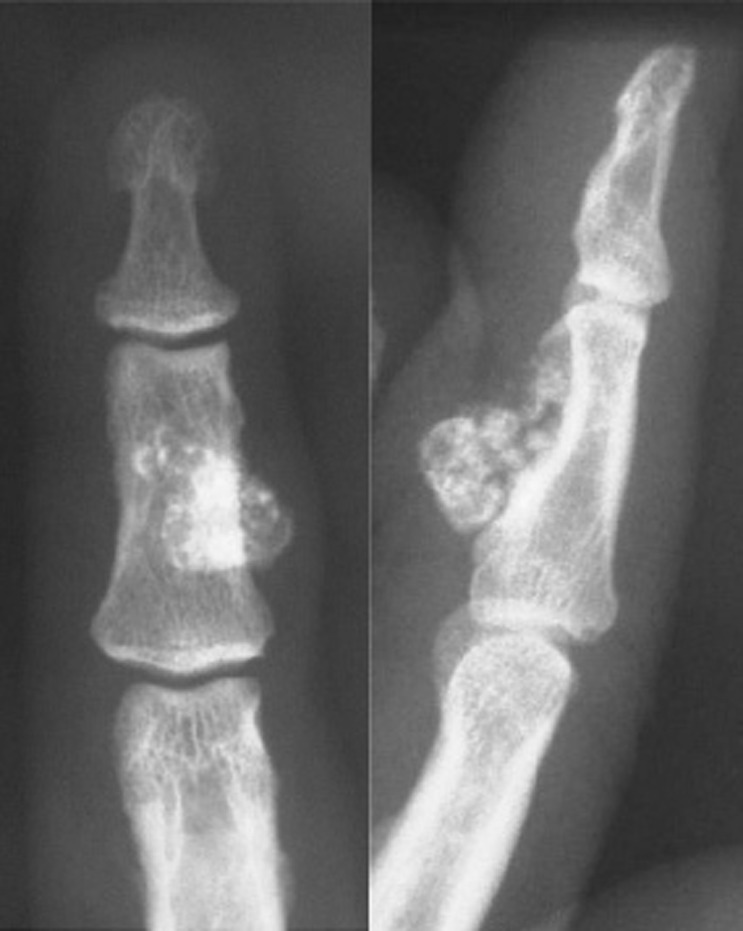
A 52-year-old man presenting with a 5-year history of mass on the palmar aspect of the right index finger. Anteroposterior and lateral radiographs of the right index show a juxta-cortical calcified lesion of the middle phalanx with cortical erosion.

Antero posterior and lateral radiographs of the right index finger demonstrated a dense mass approximately 1.5 cm in size. The mass extended from the palmar aspect of the second phalanx. There was cortical erosion, but no abnormality of the underlying bony architecture (Fig. 1).

Case 2: A 24-year-old woman was seen for a nine-month history of a hard mass on the second phalanx of the right medius. She denied any history of injury. Antero posterior and lateral radiographs showed a well-circumscribed calcified juxtacortical mass.

**Fig. 2:**
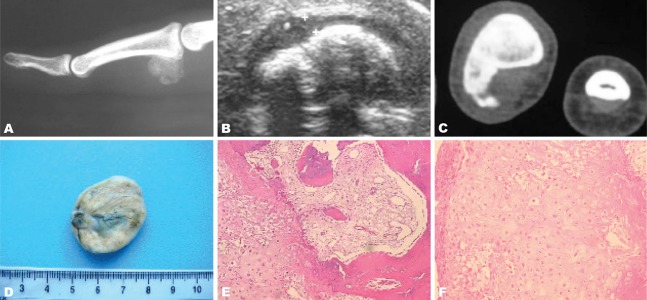
A 24-year-old woman with a nine-month history of a hard mass at the second phalanx of the right medius. A. Lateral radiography of the right medius shows a calcified well circumscribed lesion, developed at the palmar aspect of the middle phalanx base, with no adjacent bone or soft tissue abnormality. B. High resolution ultrasound of the right medius in the transversal view shows a calcified lesion surrounded by a thin hypoechoic cap. C. Transversal CT view of the right hand in bone algorithm shows a surface bone lesion with large cortical base. There is no continuity between the lesion and the underlying bone cortex. D. Gross pathology view shows a well circumscribed pedunculated mass of hard consistence and white greyish color. E. Microscopic view (HE ×200) showing a tumoral proliferation with osseous, cartilaginous and fibrous components. F. Microscopic view (HE ×400) showing a cartilaginous proliferation made of chondrocytes with irregular morphology (bizarre cells).

CT showed lack of continuity between this lesion and the medullary canal of the affected bone, but the cortex was normal (Fig. 2).

**Fig. 3:**
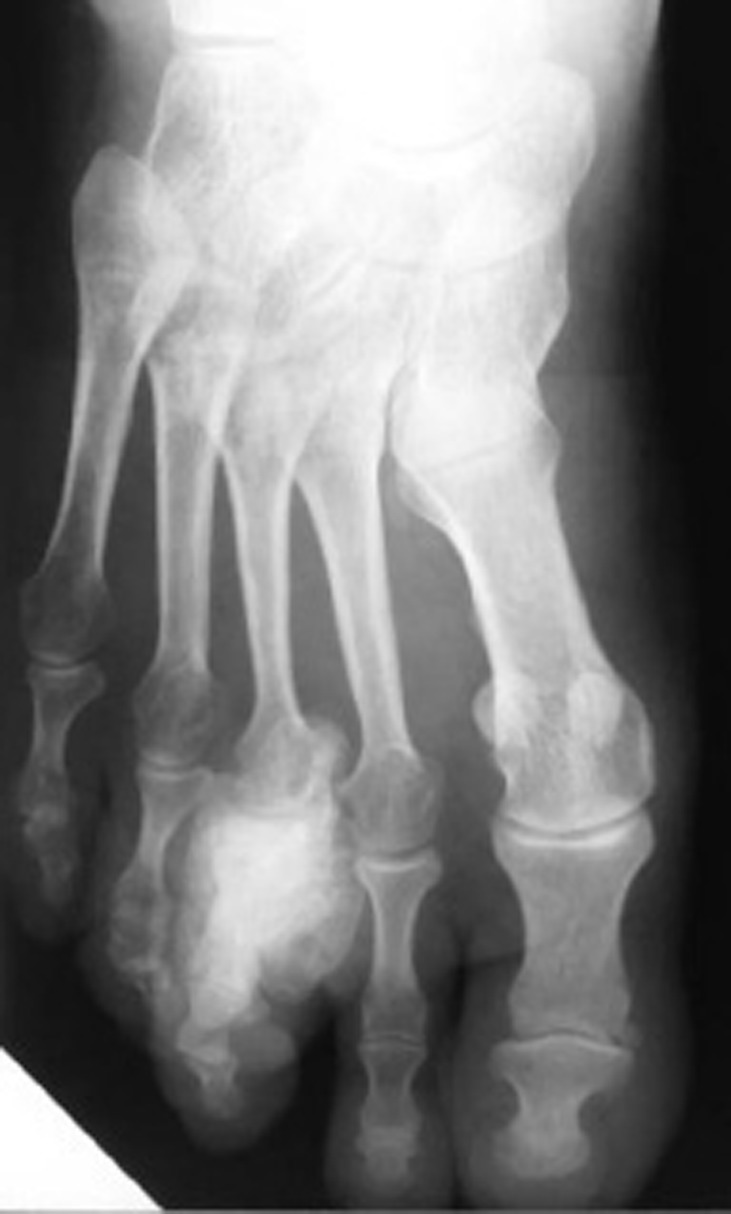
A 38-year-old woman with swelling and discomfort of the right forefoot. Antero posterior radiograph of the right foot shows an ossified mass originated from the proximal phalanx of the third toe.

Case 3: A 38-year-old woman presented with swelling and discomfort affecting her right forefoot. There was no significant past medical history nor history of trauma. On examination, there was evidence of a fusiform swelling regarding the proximal phalanx of the third right toe. Radiographs showed an ossified mass with well-defined margins and a wide base on the plantar surface of the first phalanx of the third right toe (Fig. 3).

**Fig. 4:**
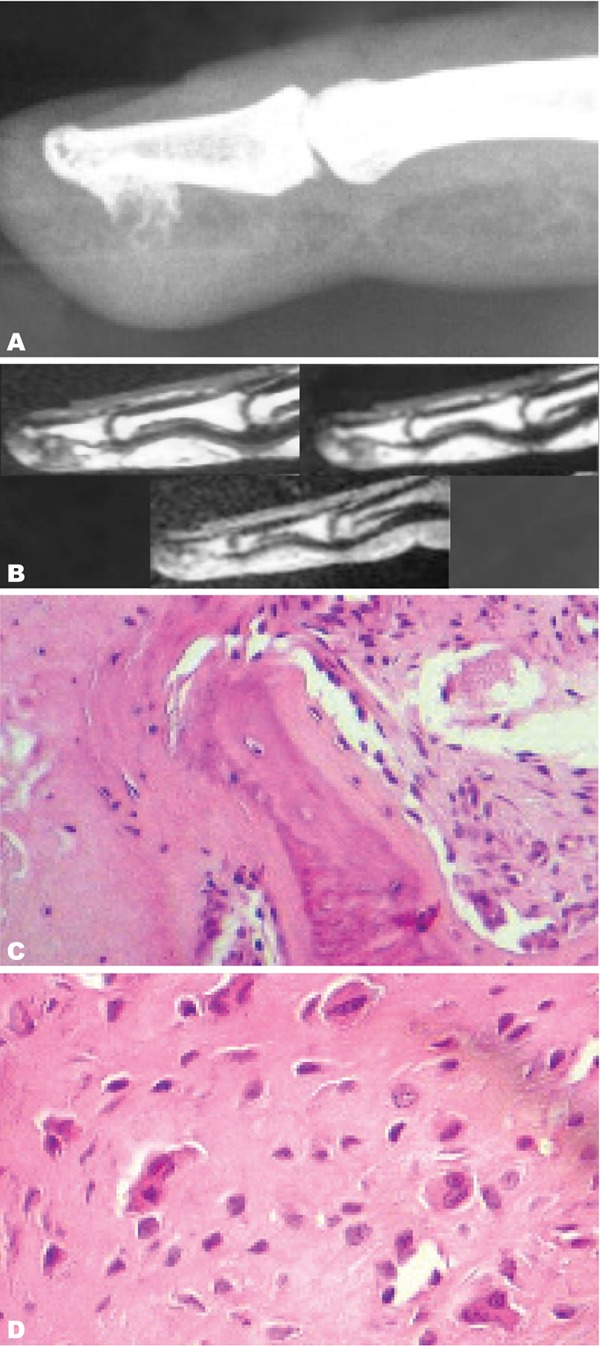
A 45-year-old woman with swelling and pain in the distal phalanx of the right medius. A. Lateral radiography of the right medius shows a calcified bone surface lesion developed from the palmar surface of the distal phalanx with soft tissue swelling but no adjacent bone abnormality. B. Sagittal MRI view of the right hand on T1W sequence, T1W sequence after intravenous Gadolinium injection and T2W sequence. The lesion shows a homogeneous low T1 signal and high T2 Signal with moderate enhancement after intravenous Gadolinium injection. C. Microscopic view (HE×200) showing bone trabecula associated with fibrous tissue. D. Microscopic view (HE×400) showing chondroid tissue made of chondrocytes of irregular size sometimes binucleated.

Case 4: A 45-year-old woman working as a tailor complained of swelling and pain of the distal phalanx of the right medius. Radiograph showed a welldefined bony mass originating from the distal phalanx of the right medius. In the T1 weighted MR image, a lesion of low signal intensity extending from the distal phalanx of the right medius was demonstrated. The cortex and bone marrow of the underlying bone were of normal signal intensity. The soft tissues showed no abnormality. T2 MR imaging of the lesion showed slightly increased signal intensity centrally with the periphery having much higher signal intensity. T1 weighted image following gadolinium administration showed uniform enhancement of the lesion (Fig. 4).

All patients underwent surgical excision of the lesions with pathological diagnostic confirmation. Two patients were lost to follow-up evaluation. Regarding the other two patients, the post operative follow-up period ranged from six to eighteen months without clinical or radiological signs of recurrence.

## Discussion

BPOP is a benign and rare surface lesion of the bone that usually affects the proximal and middle phalanges, and the metacarpal or metatarsal bones.[1][4][5] The hands are four times more commonly affected than the feet 4 and the lesion does not tend to involve the distal phalanx in distinction from subungual exostosis.[6][7][8]

BPOP is less commonly found in other sites.[5] Some unusual locations have been reported such as the humerus[9] and the clavicle.[10]

BPOP affects males and females equally.[1][5][6][7] The lesion is most common in the third and fourth decade, but there may be a wide range of age presentation.[5][6][11]

The typical clinical presentation is a painless swelling that grows over a period of months to years.[7][12] Pain or skin erythema may occasionally be experienced by the patient due to mass effect or mechanical problems.[7][11][13][14] Clinical examination usually shows a small, firm mass ranging from 0.4 to 3 cm in diameter not involving the overlying skin.[1][15][16] Joint motion may be limited when the lesion is located at the end of the bone.[17]

Radiographically, BPOP is a well-marginated, calcified or ossified mass arising directly from the cortical surface of the underlying bone.[5][15][17][18] It is generally attached by a broad base and the underlying cortex is intact.[2][5][17] There is no periosteal new bone formation. However, cortical erosion has been reported.[17][19][20]

Fine cut computed tomography (CT) scan shows a well-defined, intensely calcified or ossified mass, arising from the cortex of the affected bone. CT is better than plain radiography in showing the absence of continuity between the cortex, the medullary cavity of BPOP and the bone of origin and the absence of cortical flaring in this affected bone.[14][21][22]

On MRI images, BPOP displays homogeneous low signal intensity on T1 weighted sequences with uniform enhancement after gadolinium administration.[5][12][17] The lesion shows a high signal intensity on T2 weighted images, a slightly increased signal centrally with its periphery having higher signal intensity.^6^,^12^ Periosteal reaction and medullary involvement are typically absent in BPOP which is associated with normal underlying bone and adjacent soft tissues.[5][2][7][20][21]

While most of the authors agree about these latter MRI findings, Orui [4] reported a case of BPOP with ill defined margins and inflammatory extension to the intramedullary area and surrounding subcutaneous tissue. He explained that this discrepancy may be caused by the different osteocartilaginous maturation of BPOP.

BPOP has an atypical histological appearance, as reflected by the use of the word "bizarre" in its name. Microscopic typical findings include highly cellular, disorganized and irregular cartilage associated with proliferation of bizarre fibroblasts, disorganized bone and spindle shaped fibroblasts located in the intertrabecular space.[7][12][23][24] The bony trabecula, with uniform osteoblasts, varying in size, are unevenly calcified and are typically covered by a cartilaginous cap with irregular endochondral ossification.[7][17] This cartilaginous component of the lesion contains irregular groups of bizarre, enlarged and sometimes binucleated chondrocytes. These cells are not organized in columns and lose their own lacunar partition. The spindle cells are arranged loosely in fibrous tissue between the bony trabecula and do not show any specific pattern. Mitotic figures are often seen, but neither atypical mitosis nor cytological atypia is seen in the spindle cells.[7][8][17][23]

The presence of an unusual form of calcified cartilage that stains blue on hematoxylin and eosin (H and E) stain is characteristic. This feature, which has come to be known as "blue bone", was first noted by Meneses 2 and is found even after decalcification.[8][13][17]

BPOP can appear similar to osteochondroma, the most common benign bone tumor.[6][8][25] The latter is more likely to be found in the metaphysis of long bones. Only 4% of solitary osteochondromas involve the hand.[7][26] Radiographically osteochondromas are typically sessile, arising near the end of phalanges or metacarpal bones. They show continuity with the underlying medullary cavity of the bone of origin.[27]

The absence of continuity between the lesion and medullary cavity of the bone has been singled out as a key radiographic finding to differentiate BPOP from osteochondroma.[4][6][8][10][12][14][15][17][18][20][21][22][25][28]

Recently, Rybak et al. presented four cases of pathologically proven BPOP in which corticomedullary continuity with the underlying bone was demonstrated on imaging. He indicates that what used to be a critical radiological finding distinguishing BPOP is not a reliable method for either identifying or excluding this lesion.[11]

Grossly BPOP may also be confused with osteochondromas, but some histological differences are apparent.[2][28] In addition to the continuity between the medullary canal and cortical bone with the underlying bone, in osteochondroma, chondrocytes lack atypia and often are arranged in parallel lacunar spaces.[2][13][17][24] Moreover, osteochondromas have regular arrangement of the trabecula of the bone, oriented at ninety degrees to the cartilage cap.[16]

Parosteal osteosarcoma is very rarely found in the hands and feet. It typically appears as a dense lobulated mass attached by a broad-based pedicle to the cortex and has heavy mineralization with a sclerotic appearance.^6^,^14^ The radiographic feature that differentiates BPOP and parosteal osteosarcoma is the cortical and soft-tissue infiltration and periosteal reaction, two features absent in BPOP.[9][21] Although there has been one report of BPOP with cortical invasion and soft tissue infiltration [19], this finding is exceedingly uncommon.

Because of the increased cellularity and fibrous tissue proliferation within the bony portion, BPOP needs also to be distinguished histologically from parosteal osteosarcoma and a grade I or II chondrosarcoma.[29] In parosteal osteosarcoma, cartilage may also be present either on the surface of the lesion or deeper within the mass.[9] The cornerstone of this tumor is the spindle cell proliferation of cytologically bland-looking spindle cells and relatively mature bony trabecula.[14] In addition, severe atypia of fibrocytes, not seen in BPOP, is present.[16][29]

Peripheral chondrosarcoma, typically found in adults, is rare in the hands and feet. Radiographically, it appears as a mass with ring-like or popcorn calcification.[14]

Peripheral chondrosarcoma is histologically different from BPOP by the well-differentiated hyaline cartilage with lobular architecture and no mitosis in grade I variant. In grade II, the cellularity increases and foci of necrosis are seen.[8]

Turret exostosis or ossified hematoma is a smooth dome shaped parosteal bone proliferation related to trauma and may also mimic BPOP. It is usually located on the dorsal aspect of the proximal or middle phalanx.[6][14][27][30]

A stress fracture with extensive callous formation may as well look like BPOP. Typically, it presents as a thin lucent fracture line with periosteal reaction and cortical thickening. The diagnosis of stress fracture should be made from the history in conjunction with radiographic features.[5]

The other radiologic and histologic differential diagnoses of BPOP are florid periostitis and myositis ossificans. Florid reactive periostitis is a rare benign lesion usually affecting the small bones of the hands and feet in second and third decade patients. It is frequently related to trauma and is characterized by soft tissue swelling, variable lamellated or mature periosteal reaction, slight periosteal elevation and juxtacortical soft tissue calcification.[6][14] Histologically, cartilage is absent in florid periostitis and periosteal reaction is absent in BPOP.[16][24][31] Myositis ossificans may mimic BPOP, is rarely located in the hand and if it occurs in a juxtacortical location, it may be known as periostitis ossificans. It appears as a soft tissue mass which characteristically ossifies from the margins inwards (zoning phenomenon). It is usually separated from the adjacent bone which can make periosteal reaction.[13][14][27]

Although agreement exists with regarding most common locations of BPOP, much debate still exists about its pathogenesis. Horiguchi [7] considered BPOP as a reparative process after periosteal injury. He added that the history of trauma at the site of the lesion, which has been reported in a considerable number of patients[14][16][20][29][31], may also support this reparative theory. He also demonstrates the expression of basic fibroblast growth factor (bFGF), vascular endothelial growth factor (VEGF) and chondromoduline I (ChMI) in the cartilaginous cap of BPOP, which are all involved in osteocartilaginous formation.

These facts support the theory first published by Yuen [31] which even suggested the existence of a continuum among florid reactive periostitis, BPOP and turret exostosis and that each is a different stage of proliferative periosteal process.

Ly et al.[20] proposes a relationship between myositis ossificans and BPOP and was the first to report a case in which trauma with subsequent development of myositis ossificans later progressed to BPOP. This fact supports the theory that minor trauma is an etiologic factor in the development of BPOP, but in this same case the diagnosis of myositis ossificans was made radiographically with no histological confirmation.

These theories discussed earlier are based on a history of trauma initial to BPOP, whereas, many patients do not complain about previous injury or trauma. In addition, in the series by Meneses [2], there was a history of trauma in only 30% of cases.

Orui^4^ reported a patient with BPOP that occurred 2 years after bilateral leg erythema nodosum. Systemic or focal inflammation might have been responsible in his opinion.

Recently, Nilsson [32] reported a translocation between chromosome 1 and 17. By cytogenetic analysis of five patients with BPOP, he showed a balanced translocation t (1;17) (q32;q21). This translocation seems to be a recurrent and pathogenetically significant aberration in BPOP. In addition, through database searches, Nilsson [32] identified a number of genes located within the 2 sequences spanning the breakpoints of chromosomes 1 and 17. These are BRCA1, which is associated with breast cancer, and COL1A1, which is involved in dermatofibrosarcoma protuberans and giant cell fibroblastoma. Thus, the present data do not indicate direct involvement of any of these 2 tumorassociated genes in BPOP development. These recent findings suggest that BPOP represents a neoplastic rather than a reactive lesion.

BPOP is a benign lesion; therefore no treatment is required if it is asymptomatic.[12] If the patient is symptomatic (pain or compromised function), the definitive treatment is surgical excision with wide margins.[12][15][22][23][32][33] Following superficial dissection, Nora’s lesion is well demarcated and appears as a bony mass, covered by a cartilage cap with an overlying pseudocapsule.[17] En bloc negative margin excision by excision of the pseudocapsule over the lesion and any periosteal tissue beneath the lesion and decortication of any areas in the underlying host bone that appear abnormal has been shown to be beneficial in preventing local recurrence.[17][30]

In fact, BPOP has a remarkable tendency to recur.[9][16][21][23][30] Nora [1] presented 18 (51%) local recurrences among 35 cases of BPOP. Meneses [2] reported a recurrence rate of 55% in a series of 65 patients and almost half have had a second recurrence. The time interval between excision and local recurrence ranges from 2 months to 2 years.[1][2][29]

Ly et al.[20] found that recurrence is common when a BPOP lesion shows increased uptake on bone scintigraphy. He consequently recommended to perform bone scan prior to surgery, then to delay surgery until the lesion is no longer active.

Despite this high recurrence rate and atypical histological appearance, no malignant transformation, metastases or deaths have been described so far in patients with BPOP.[1][2][9][16]

Because of the high local recurrence rate and lack of adjuvant therapy options, Nora's lesion requires long-term follow-up.[18][30] During the first year of follow up, it is recommended to review patients every 6 months.[15] Following this, further reviews should be performed yearly for the next 2 years.[17] However, the patient must be informed of the potential for further recurrence at a later date.

## References

[1] Nora FE, Dahlin DC, Beabout JW (1983). Bizarre parosteal osteochondromatous proliferations of the hands and feet.. Am J Surg Pathol.

[2] Meneses MF, Unni KK, Swee RG (1993). Bizarre parosteal osteochondromatous proliferation of bone (Nora's lesion).. Am J Surg Pathol.

[3] Chaabane S, Chelli Bouaziz M, Ben Ghars K, Abid L, Jaafoura MH, Ladeb MF (2010). A lesion of juxtacortical origin.. Tunis Orthop.

[4] Orui H, Ishikawa A, Tsuchiya T, Ogino T (2002). Magnetic resonance imaging characteristics of bizarre parosteal osteochondromatous proliferation of the hand: a case report.. J Hand Surg Am.

[5] Kenan S, Abdelwahab IF, Klein MJ, Hermann G, Lewis MM (1993). Lesion of juxtacortical origin (surface lesions of bone).. Skeletal Radiol.

[6] Torreggiani WC, Munk PL, Al-Ismail K, O'Connell JX, Nicolaou S, Lee MJ (2001). MR imaging features of bizarre parosteal osteochondromatous proliferation of bone (Nora's lesion).. Eur J Radiol.

[7] Horiguchi H, Sakane M, Matsui M, Wadano Y (2001). Bizarre parosteal osteochondromatous proliferation (Nora's lesion) of the foot.. Pathol Int.

[8] Bovee JV, Hogendoorn PCW (2001). Cartilage-forming tumours of bone and soft tissue and their differential diagnosis.. Current Diagnostic Pathology.

[9] Bush JB, Reith JD, Meyer MS (2007). Bizarre parosteal osteochondromatous proliferation of the proximal humerus: case report.. Skeletal Radiol.

[10] Vlychou M, Gibbons CL, Rigopoulou A, Ostlere SJ, Athanasou NA (2008). Bizarre parosteal osteochondromatous proliferation of the clavicle.. J Shoulder Elbow Surg.

[11] Rybak LD, Abramovici L, Kenan S, Posner MA, Bonar F, Steiner GC (2007). Cortico-medullary continuity in bizarre parosteal osteochondromatous proliferation mimicking osteochondroma on imaging.. Skeletal Radiol.

[12] Rastogi R (2007). Musculoskeletal radiology Quiz Answers.. Indian J Radiol Imaging.

[13] Shin BK, Cho HD, Yum BW, Choi JS, Kim CH (1999). Bizarre parosteal osteochondromatous proliferation of the femur (Nora’s lesion)- a case report.. J Korean Bone Joint Tumor Soc.

[14] Bandiera S, Baccini P, Bertoni F (1998). Bizarre parosteal osteochondromatous proliferation of bone.. Skeletal Radiol.

[15] Walsh JC, Murphy D, Freihaut RB, O’Keane JC, Stephens MM (2006). Bizarre parosteal osteochondromatous proliferation of the fifth metatarsal (Nora’s lesion)-Case report.. Foot Ankle Surg.

[16] de Lange EE, Pope TL, Fechner RE, Keats TE (1987). Case report 428: Bizarre parosteal osteochondromatous proliferation (BPOP).. Skeletal Radiol.

[17] Michelsen H, Abramovici L, Steiner G, Posner MA (2004). Bizarre parosteal osteochondromatous proliferation (Nora’s lesion) in the hand.. J Hand Surg Am.

[18] Lam FCY, Tsang TK, Fung HS, Wai MW, Shu SJ (2006). Bizarre parosteal osteochondromatous proliferation (Nora's disease).. Hong Kong J Emerg Med.

[19] Helliwell TR, O’Connor MA, Ritchie DA, Feldberg L, Stilwell JH, Jane MJ (2001). Bizarre parosteal osteochondromatous proliferation with cortical invasion.. Skeletal Radiol.

[20] Ly JQ, Bui-Mansfield LT, Taylor DC (2004). Radiologic demonstration of temporal development of bizarre parosteal osteochondromatous proliferation.. Clin Imaging.

[21] Soon JL, Chang HC, Sim CS, Teoh LC, Low CO (2003). A case of Bizarre Parosteal Osteochondromatous Proliferation of the hand.. Singapore Med J.

[22] Efstathopoulos NE, Papagelopoulos PJ, Lazarettos IT, Savvidou OD, Kaseta MA, Giannakou N (2005). Bizarre parosteal osteochondromatous proliferation of the second metatarsal bone (Nora's lesion).. Orthopedics.

[23] Pillai A, Shenoy R, Ried R, Tansey P (2006). Bizarre Parosteal Osteochondromatous Proliferation of the hand.. J Bone Joint Surg Br.

[24] De Smet L, Lambert I, Sciot R (2001). Bizarre parosteal osteochondromatous proliferation of the hand: report of two cases.. Chir Main.

[25] Boussouga M, Harket A, Bousselmane N, Lazrak K (2008). Bizarre parosteal osteochondromatous proliferation (Nora’s lesion) of the forefoot.. Acta Orthop Belg.

[26] Unni KK (1996). Dahlin's bone tumors: general aspects and data on 11087 cases.

[27] James SLJ (2006). Surface lesions of the bones of the hand.. Eur Radiol.

[28] Hemmadi SS, Patel BR (1992). Bizarre parosteal osteochondroma in the foot (a case report).. J Postgrad Med.

[29] Twiston Davies CW (1985). Bizarre parosteal osteochondromatous proliferation in the hand. A case report.. J Bone Joint Surg Am.

[30] Gruber G, Giessauf C, Leithner A, Zacherl M, Clar H, Bodo K (2008). Bizarre parosteal osteochondromatous proliferation (Nora lesion): a report of 3 cases and a review of the literature.. Can J Surg.

[31] Yuen M, Friedmann L, Orr W, Cockshott WP (1992). Proliferative periosteal processes of phalanges: a unitary hypothesis.. Skeletal Radiol.

[32] Nilsson M, Domanski HA, Mertens F, Mandahl N (2004). Molecular cytogenetic characterization of recurrent translocation breakpoints in bizarre parosteal osteochondromatous proliferation (Nora's lesion).. Hum Pathol.

[33] Le Bellec Y, Asfazadourian H (2005). Bizarre parosteal osteochondromatous proliferation (Nora's lesion). Two case reports.. Chir Main.

